# Three-dimensional Cardiomyocytes Structure Revealed By Diffusion Tensor Imaging and Its Validation Using a Tissue-Clearing Technique

**DOI:** 10.1038/s41598-018-24622-6

**Published:** 2018-04-27

**Authors:** Sang-Eun Lee, Christopher Nguyen, Jongjin Yoon, Hyuk-Jae Chang, Sekeun Kim, Chul Hoon Kim, Debiao Li

**Affiliations:** 1 Division of Cardiology, Severance Cardiovascular Hospital, Yonsei University College of Medicine, Yonsei University Health System, Seoul, 03722 South Korea; 2 Integrative Cardiovascular Imaging Center, Yonsei University Health System, Seoul, 03722 South Korea; 3 Biomedical Imaging Research Institute, Cedars-Sinai Medical Center, Los Angeles, CA 90048 USA; 4 Cardiovascular Research Center, Massachusetts General Hospital, Charlestown, MA 02129 USA; 5 Harvard Medical School, Boston, MA 02115 USA; 6 Departement of Pharmacology, Yonsei University College of Medicine, Yonsei University Health System, Seoul, 03722 Korea; 7 Graduate School of Biomedical Engineering, Yonsei University College of Medicine, Seoul, 03722 South Korea

## Abstract

We characterized the microstructural response of the myocardium to cardiovascular disease using diffusion tensor imaging (DTI) and performed histological validation by intact, un-sectioned, three-dimensional (3D) histology using a tissue-clearing technique. The approach was validated in normal (n = 7) and ischemic (n = 8) heart failure model mice. Whole heart fiber tracking using DTI in fixed *ex-vivo* mouse hearts was performed, and the hearts were processed with the tissue-clearing technique. Cardiomyocytes orientation was quantified on both DTI and 3D histology. Helix angle (HA) and global HA transmurality (HAT) were calculated, and the DTI findings were confirmed with 3D histology. Global HAT was significantly reduced in the ischemic group (DTI: 0.79 ± 0.13°/% transmural depth [TD] and 3D histology: 0.84 ± 0.26°/%TD) compared with controls (DTI: 1.31 ± 0.20°/%TD and 3D histology: 1.36 ± 0.27°/%TD, all *p* < 0.001). On direct comparison of DTI with 3D histology for the quantitative assessment of cardiomyocytes orientation, significant correlations were observed in both per-sample (R^2^ = 0.803) and per-segment analyses (R^2^ = 0.872). We demonstrated the capability and accuracy of DTI for mapping cardiomyocytes orientation by comparison with the intact 3D histology acquired by tissue-clearing technique. DTI is a promising tool for the noninvasive characterization of cardiomyocytes architecture.

## Introduction

The left ventricle contains laminar helical structures positioned in a left-handed (epicardium) to right-handed (endocardium) orientation^1,2^, which is a crucial determinant of both the mechanical and electrical function of the heart^3,4^. Visualization and quantitative analysis of this complex, three-dimensional (3D) microstructure could improve the understanding of heart-structure relationships in normal development and cardiovascular disease. Nevertheless, this underlying cardiomyocytes architecture has mostly been inferred using invasive biopsies or methods that rely on destructive histological sectioning^1,5^.

Imaging techniques such as diffusion tensor imaging (DTI) made it possible to directly visualize these tissue microstructure of the heart^6–8^. Recent technological advances have also enabled the application of DTI to a beating human heart, allowing for clinical applications^8–10^. However, the ability of DTI to delineate the tissue microstructure of the heart has not been fully validated through direct comparison with 3D non-destructive histological analysis of an intact heart, hence limiting its clinical application.

Tissue-clearing techniques^11,12^, which allow the molecular phenotyping and imaging of intact tissues, may address the current limitations of studying the heart microstructure and histologically validating cardiac DTI. These techniques clear the intact tissue of light-scattering lipids^13^. Because both connective tissue and cardiomyocytes are aligned in the same direction, forming a laminar structure^2^, simple auto-fluorescence of non-specific myocardial proteins can directly reveal cardiomyocytes orientation, the same vector calculated by DTI. Therefore, tissue-clearing techniques can be used to validate the accuracy of DTI for assessing cardiac microstructure.

We aimed to validate the integrity of DTI using CLARITY, a tissue-clearing technique^11^, in intact, normal mouse hearts to confirm whether microstructural information acquired by DTI is comparable to that provided by optical imaging of a cleared, intact heart.

## Results

### Validation of the tissue-clearing technique

**Figure 1:**
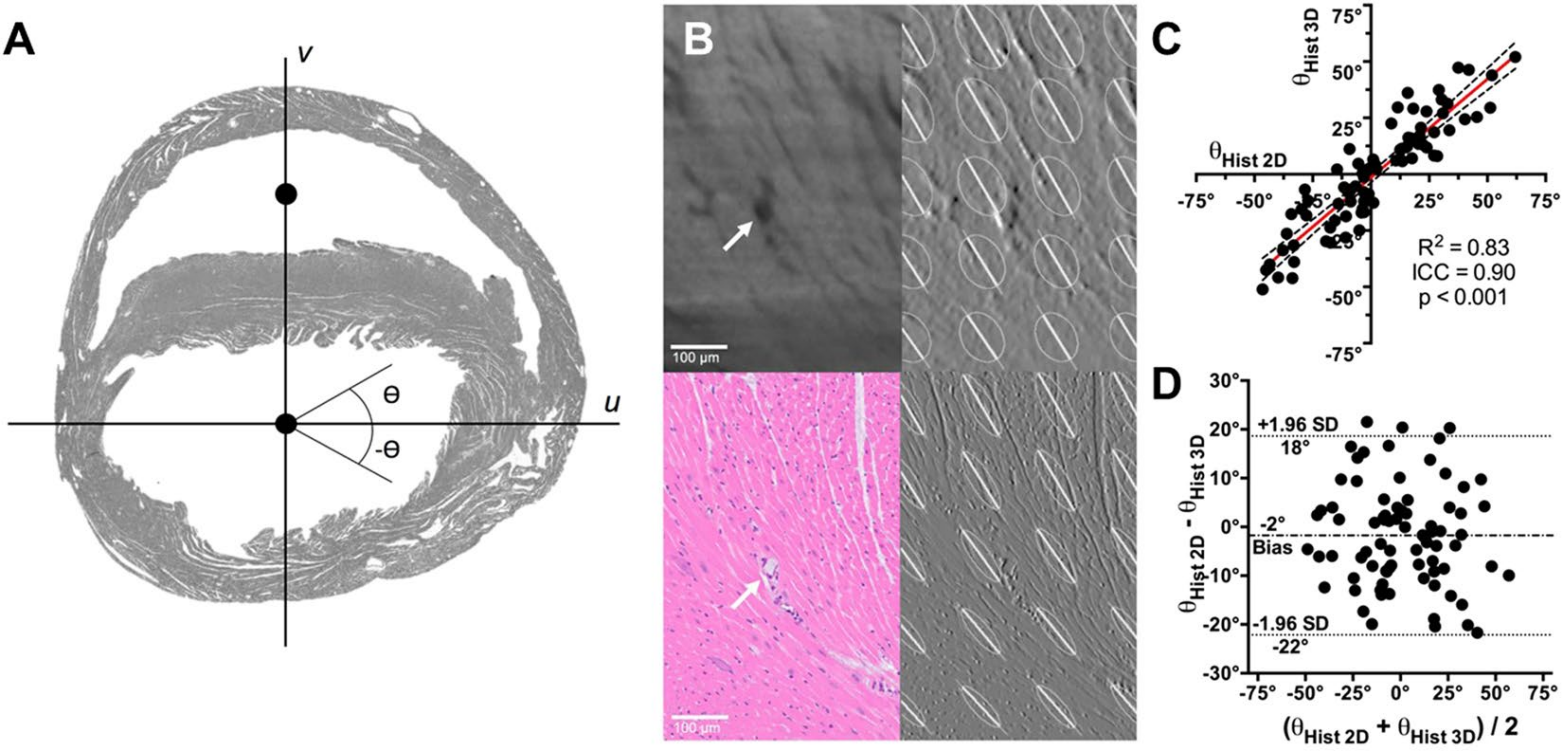
Comparison of in-plane cardiomyocytes angles, Ɵ, calculated from mirror sections using conventional 2-dimensional (2D) histology and 3-dimensional (3D) histology with a tissue-clearing technique. (**A**) Determination of the axes, *u* and *v*, used to calculate Ɵ. Axis *v* is defined as the vector containing the centers of mass of the left and right ventricles. Axis *u* is orthogonal to *v*. (**B**) Zoomed-in section showing the cardiomyocytes delineation on 3D histology (upper panels) and 2D histology (lower panels). In addition to cardiomyocytes structures, a blood vessel (white arrow) can be visualized by both techniques (left column). Calculated 2D structure tensors are overlaid on the image gradient maps *f*_*u*_, of both 2D and 3D histology methods, demonstrating similar cardiomyocytes orientation (right column). (**C**) Correlation and (**D**) Bland-Altman plots demonstrate significant (*p* < 0.001) correlation (R^2^ = 0.83) and agreement (ICC = 0.90), with insignificant negative bias (−2° ± 10°, [−22° to 18°]), between the 2D and 3D histology methods.

The in plane cardiomyocytes angle calculated from the optical image showed no difference from the in-plane cardiomyocytes angle calculated from the section stained with H&E (Fig. 1). There was also a significant correlation (R^2^ = 0.83, *p* < 0.001) and substantial agreement (ICC = 0.90) in the calculated in-plane cardiomyocytes angle between 3D tissue-clearing histology and gold-standard 2D histology with H&E staining. A non-significant negative bias (−2° ± 10°, [−22° to 18°]) was found, and the Bland-Altman plot revealed no systematic (Type I or II) errors.

### Intact adult mouse heart 3D optical imaging

**Figure 2:**
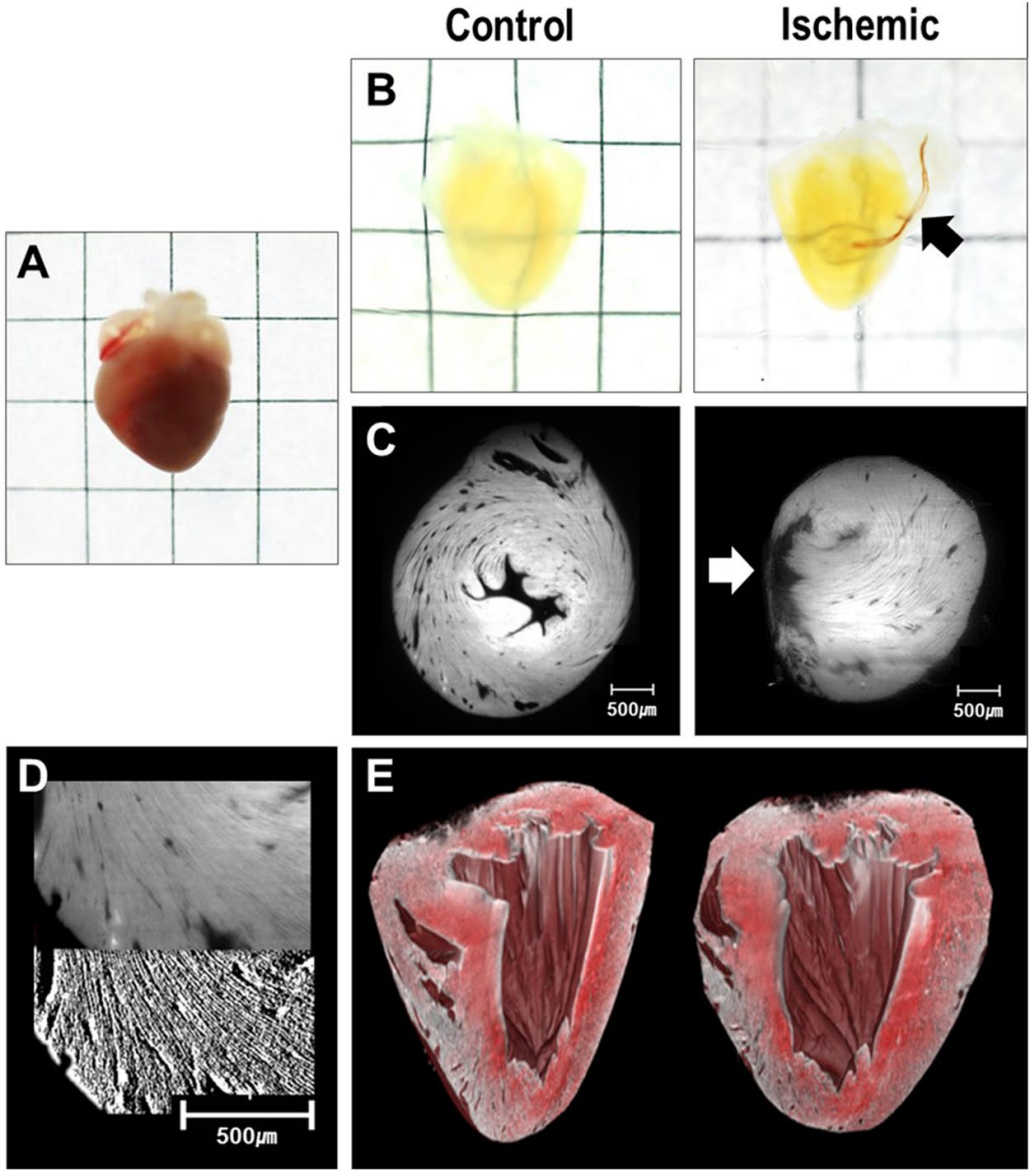
Representative figure of tissue-cleared hearts and light-sheet images. (**A**) Sample of a mouse heart before tissue-clearing process (**B**) Cleared heart samples of control (left) and ischemic hearts (right). Suture used for the permanent ligation of left anterior descending artery can be seen (black arrow) (**C**) Light-sheet image of control (left) and ischemic (right) hearts. Infarcted area with thinning is observed (white arrow) (**D**) Image processing of raw light-sheet images (upper panel) using Sobel filters (lower panel) (**E**) 3D reconstruction of the entire heart from light-sheet images.

The tissue-clearing technique was applied in seven normal and eight ischemic adult mouse hearts. Echocardiography at the day of sacrifice revealed reduced left ventricular systolic function in ischemic hearts, compared with controls (M-mode fractional shortening: 23.1 ± 3.2% vs. 52.7 ± 7.6%, *p* < 0.001). Figure 2 shows the effectiveness of the tissue-clearing technique to remove light-scattering lipids from murine heart tissue. Myocardial laminar structures about 15−20 µm in size could be directly visualized with the endogenous auto-fluorescence of myocardial tissue. An additional movie file shows this in more detail [see Video 1].

**Figure 3:**
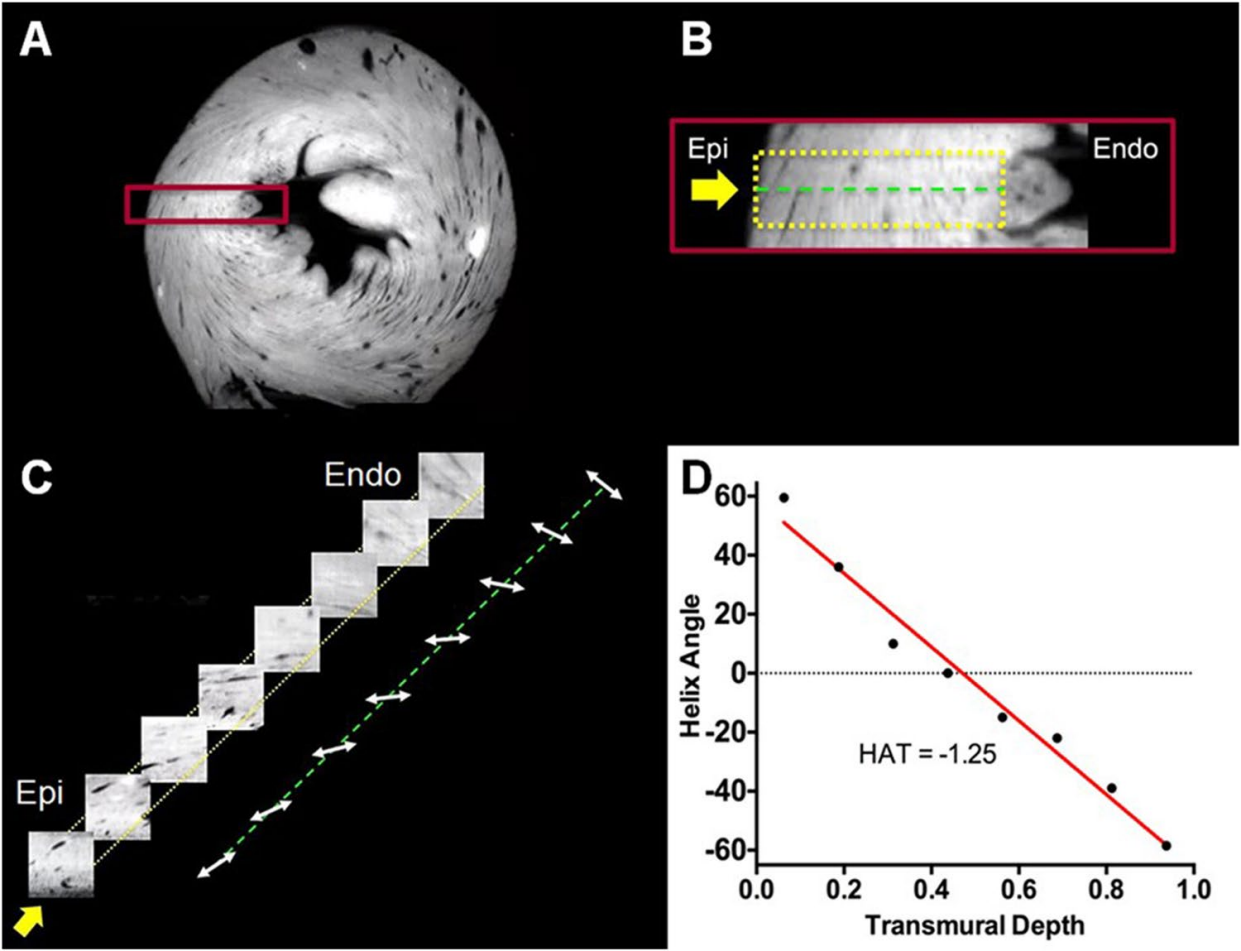
(**A**) Short-axis view of a normal heart from 3D optical raw data. (**B**) Zoomed-in region (red box) defining the epicardium (epi) and endocardium (endo) boundaries (dashed yellow box) of the virtual transmural sectioning. Orientation of the virtual transmural sectioning (yellow arrow) is orthogonal to the epicardium surface. The center line (green dotted line) of the virtual sectioning and the longitudinal axis (base to apex) are used as reference lines to calculate the helix angle. (**C**) Each virtual transmural sectioning block (8 total) undergoes structure tensor analysis to calculate the myocardial cardiomyocytes orientation. The calculated orientations are placed on the center (green dotted) line. (**D**) Helix angle vs. transmural depth plotted from the calculated virtual transmural sectioning blocks.

Cardiomyocytes twisted around the blood pool in the short-axis plane, with epicardium and endocardium having a significant through-plane component consistent with a helical structure. This was confirmed by transmural virtual sectioning of the same optical image (Fig. 3) exhibiting the presence of right-handed helically oriented layers smoothly transitioning to a left-handed helical orientation from the endocardium to epicardium. HAs were quantified and plotted against transmural depth to further demonstrate the transmural transition of right-handed to left-handed helical orientation.

### Intact adult mouse heart DTI imaging

**Figure 4:**
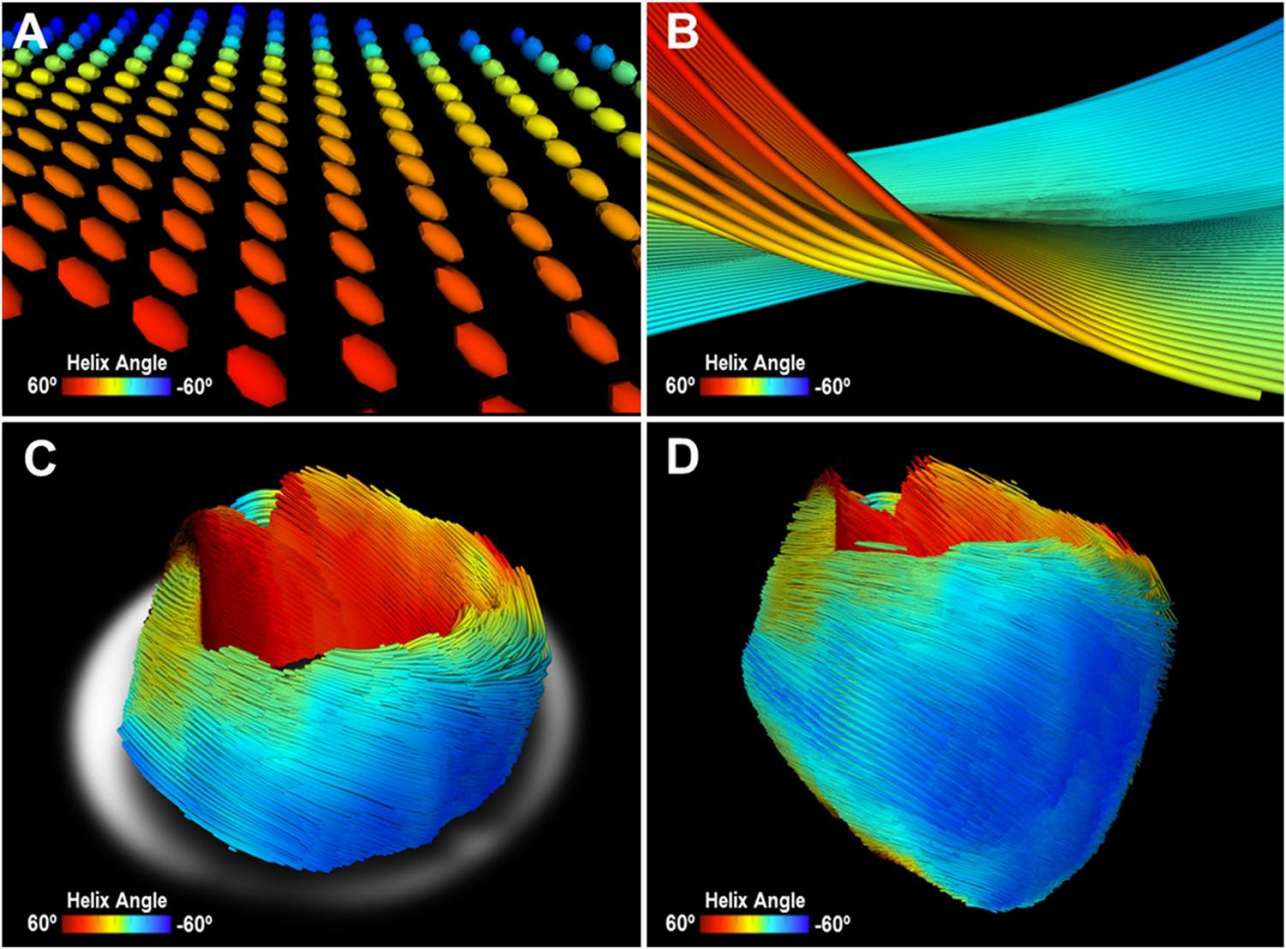
Representative image of mouse heart DTI. (**A**) Zoomed-in view of 3D diffusion tensors calculated at each voxel in the short-axis plane. Orientation of the anisotropy of the diffusion tensor is conventionally assumed to be parallel to the underlying cardiomyocytes orientation. Diffusion tensors are color-coded based on the calculated helix angle. (**B**) Zoomed-in view of 3D cardiomyocytes tracking in the myocardium. (**C**) Reconstructed cardiomyocytes above the mid-left ventricle on a short-axis plane. (**D**) 3D tractography of the helix angle of a mouse heart visualizes the helical structure of the cardiomyocytes twisting around the left ventricle.

At each voxel, DTI images were reconstructed to model the self-diffusion tensor, and cardiomyocytes orientation was assumed as parallel to the primary eigenvector of the tensor (Fig. 4A). Similar to optical imaging of the cleared myocardium, DTI also qualitatively revealed cardiomyocytes twisting around the blood pool in the short-axis plane, with significant through-plane directionality in the endocardium and epicardium layers (Fig. 4). The intra-class correlation (ICC) for inter-variability of DTI-determined HA was 0.850 (*p* < 0.001).

### Comparison of optical imaging-based and DTI-based HA

**Figure 5:**
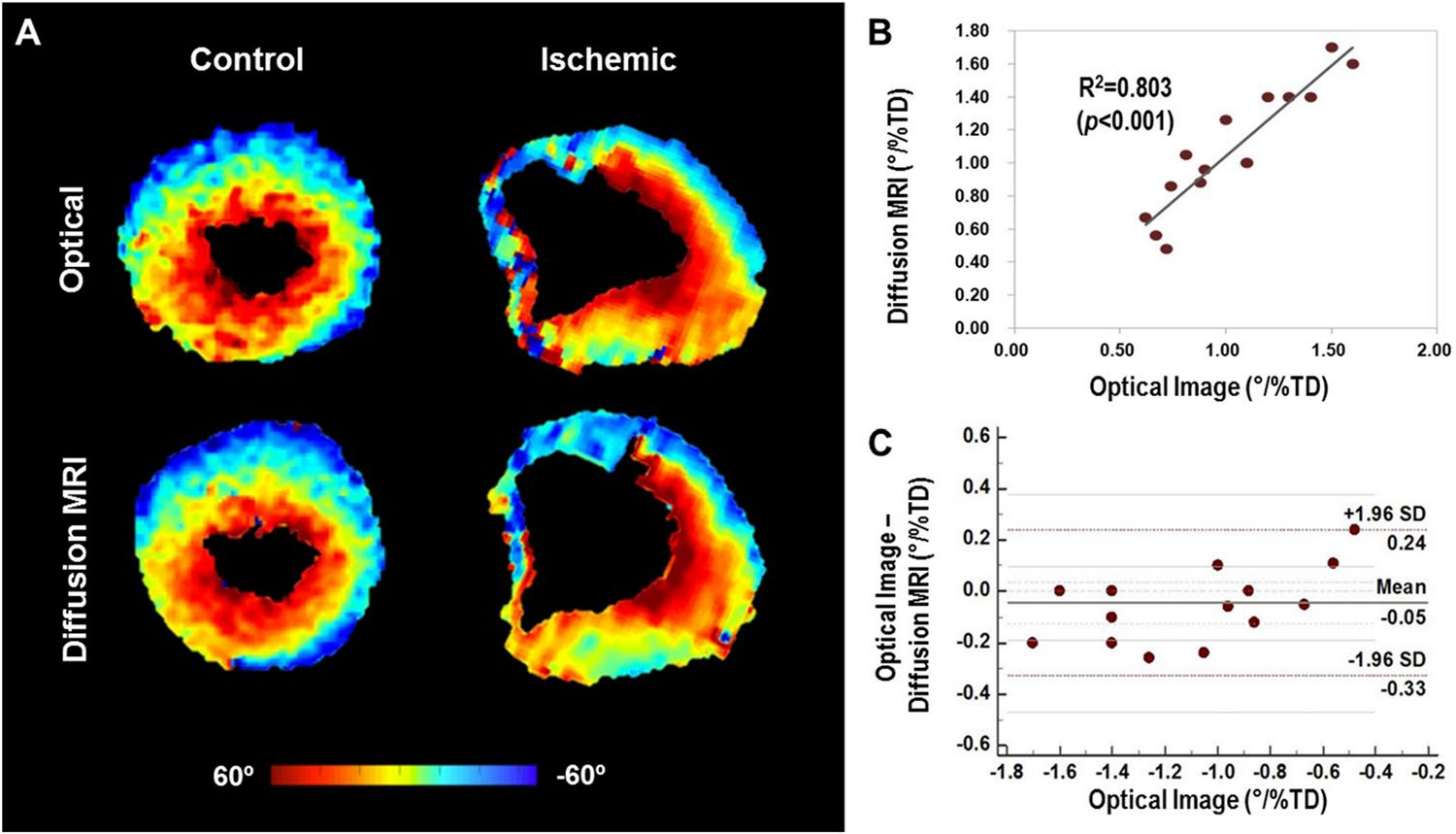
Representative helix angle maps of control and ischemic mouse hearts from optical imaging and DTI. (**A**) Helix angle transmurality of the control and ischemic groups revealed significant reduction in overall helical structure in diseased tissue (DTI: 0.79 ± 0.13°/% transmural depth [TD] and optical imaging: 0.84 ± 0.26°/%TD), compared with control tissue (DTI: 1.31 ± 0.20°/% TD and optical imaging: 1.36 ± 0.27°/%TD), specifically in the endocardium (all *p* < 0.001). (**B**) Linear correlation analysis demonstrating significant correlation (*R*^2^ = 0.803) between DTI and optical images. (**C**) Bland-Altman plot demonstrating similarity between the optical imaging-based and DTI-based cardiomyocytes helix angle transmurality estimation (Bland-Altman limits of agreement, −0.33 to 0.24°/% transmural depth [TD]).

Figure 5A shows representative short-axis HA maps of control and ischemic mouse hearts. For both imaging modalities, smooth transmural transition of the HA from epicardium to endocardium was observed in controls, while perturbed transition was observed near the infarct area in ischemic hearts. Control hearts exhibited greater helical winding, with a significantly higher magnitude of global HAT than ischemic hearts (DTI: 1.31 ± 0.20°/%TD vs. 0.79 ± 0.13°/%TD and optical imaging: 1.36 ± 0.27°/%TD vs. 0.84 ± 0.26°/%TD, all *p* < 0.001).

Significant agreement between optical imaging-based and DTI-based global HAT (R^2^ = 0.80, *p* < 0.001) was observed across all heart samples. Both control and ischemic groups also demonstrated significant agreement (R^2^ = 0.809 and R^2^ = 0.702, respectively, all *p* < 0.05) between optical imaging-based and DTI-based HAT (Fig. 5B). The Bland-Altman limits of agreement (95% confidence interval) for the difference in HAT between DTI and optical imaging were −0.33 to 0.24°/%TD for all samples (controls, −0.29 to 0.21°/%TD; ischemia, −0.38 to 0.28°/%TD), with bias of −0.05°/%TD for all samples (controls, −0.04°/%TD; ischemia, −0.05°/%TD) (Fig. 5C).

### Regional analysis

**Figure 6:**
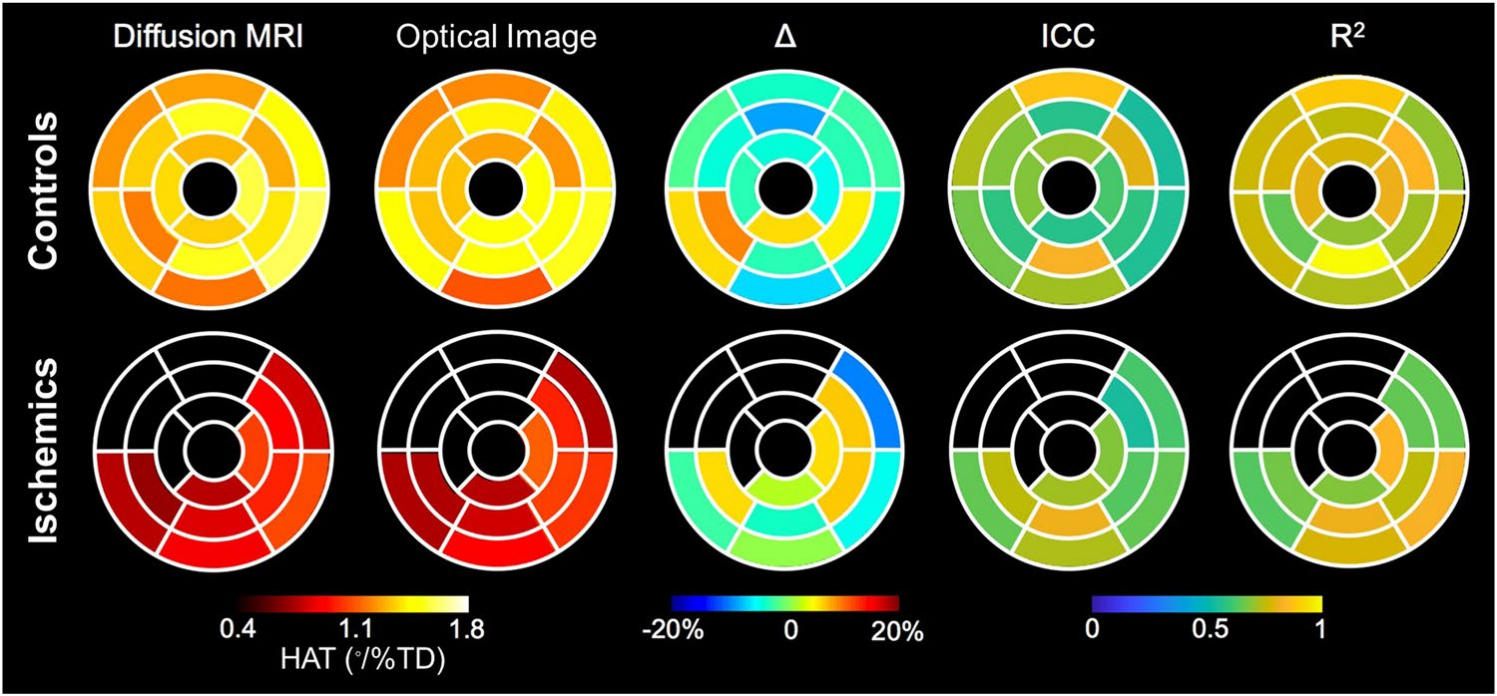
Comparison of segmental analysis between DTI and optical images in controls (upper) and ischemic group (lower). From the left, helix angle transmurality (HAT) of each 16-segments calculated in DTI, segmental HAT by optical image, differences (∆) between HAT in DTI and optical images, intraclass correlation (ICC) between DTI and optical images, and correlation coefficient (R^2^) between two measurements. Overall, DTI exhibited good segmental correlation with optical images in both controls and ischemic hearts.

Overall, excellent correlation between the two imaging modalities was observed in regional analysis based on AHA segmentation in both controls and ischemics (R^2^ = 0.725 and 0.720, respectively, and ICC = 0.710 and 0.720, respectively, all *p* < 0.05) (Fig. 6).This correlation between the two modalities was maintained in the radial segmentation, based on both linear regression analysis (all samples, R^2^ = 0.872; controls, 0.850; ischemia, 0.663, all *p* < 0.001) and Bland-Altman limits of agreement (all samples, −0.277 to 0.277°/%TD; controls, −0.231 to 0.231; ischemia, −0.333 to 0.333) with bias of −0.054°/%TD for all samples (controls, 0.059°/%TD; ischemia, 0.047°/%TD).

**Figure 7:**
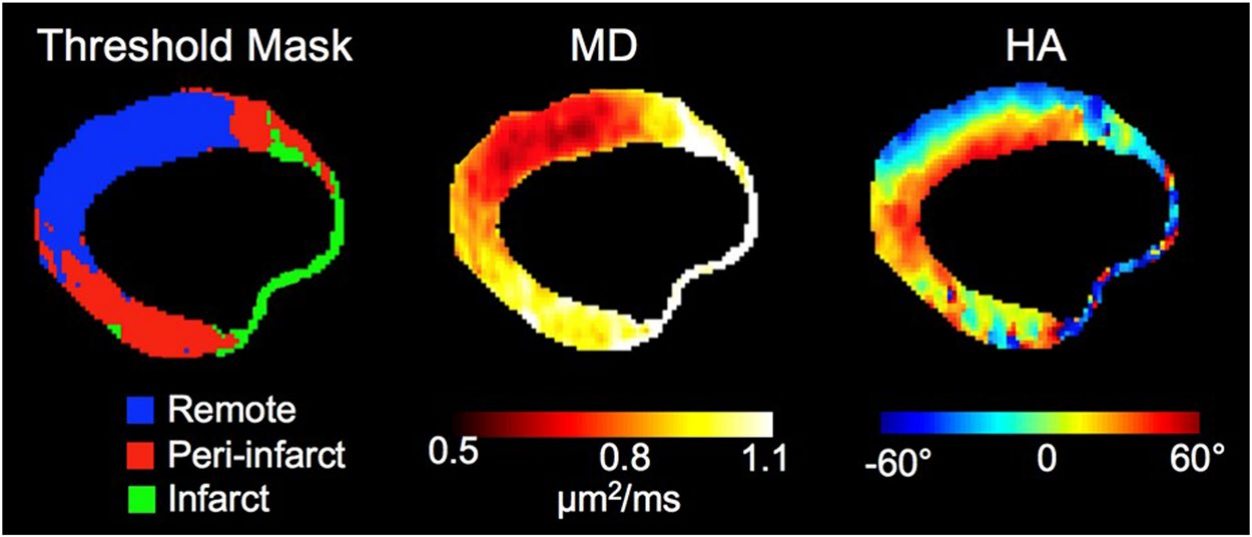
Representative figure of ischemic hearts, showing threshold mask for infarct, per-infarct, and remote areas. HA, helix angle; MD, mean diffusivity.

In ischemic hearts, peri-infarct areas, demarcated based on previously reported MD threshold, exhibited more perturbed HA, compared with remote areas, in both DTI and optical images (−0.70 ± 0.14° vs. −0.89 ± 0.14°, and −0.69 ± 0.21° vs. −0.97 ± 0.27°, respectively, all *p* < 0.05) (Fig. 7). HA values in ischemic samples were significantly decreased, compared with controls, when even remote areas were considered in both methods (DTI: −0.89 ± 0.14° vs. −1.31 ± 0.20°, optical images: −0.97 ± 0.27° vs. −1.36 ± 0.27°, respectively, all *p* < 0.05). Correlation between DTI and optical images was maintained at both per-infarct and remote areas (R^2^ = 0.857 and 0.778, respectively, all *p* < 0.05).

## Discussion

Our results confirms the integrity of DTI for quantitatively assessing the 3D microstructure of the myocardium, as validated by direct visualization of cardiomyocytes orientation with optical images obtained using a tissue-clearing technique. We were also able to reconstruct the intact 3D microarchitecture of the myocardium by DTI. When comparing in-plane cardiomyocytes angles between a pair of mirror section images, the results of optical images from the tissue-clearing technique demonstrated significant correlation and agreement with 2D histology upon H&E staining. This finding demonstrates that cardiomyocytes orientation is preserved during the tissue-clearing process, suggesting that this method can be used to validate the findings of DTI.

The histological validation of DTI has been limited because DTI-based cardiomyocytes orientations reflect 3D microstructure, while conventional histology requires destructive sectioning to acquire 2D optical images^14^. Previous researches have primarily used inherently tissue-destructive conventional techniques, such as serial histological sections, which limit analysis to small heart volumes^15^. The sectioning process may fundamentally modify or damage the cardiomyocytes microstructure, casting doubt on whether conventional 2D histology is an appropriate reference for DTI^16,17^. A recent study demonstrated the integrity of DTI using structure tensor synchrotron radiation imaging data^18^; however, validation was undertaken only in a single sample and have not applied in a diseased model. The results of current study further strengthen the integrity and accuracy of DTI by comparing DTI with optical images from intact hearts in both normal and diseased animal models.

In the present study, a tissue-clearing technique was successfully applied to intact LVs, revealing 3D cardiomyocytes orientation in its intact form. The 3D heart microstructure has rarely been directly visualized due to the complex architecture of myocardial tissue, technical limitations of previous imaging modalities, and the destructive techniques traditionally used to examine it. Identification of 3D cardiomyocytes orientation using conventional approaches cannot provide the exact vector of the cardiomyocytes, because the data must be reconstructed from images of thin slices prepared by destructive sectioning that can disrupt the myocardial microstructure, with cardiomyocytes angles only identifiable in tangential sections^19^. With the use of a tissue-clearing technique, the cardiomyocytes architecture could be visualized in an optically transparent intact heart, without destructive tissue sectioning or disturbing its original orientation.

Ours findings also demonstrated the perturbation of cardiomyocytes architecture in the presence of ischemic disease. Because myocardial structure is intimately linked to heart function, both change considerably as disease progresses. The disruption of myocardial structure has been indirectly assessed through detection of scar tissue manifested by myocardial thinning or late gadolinium enhancement on MRI^20,21^. The present study illustrated that both 3D histology and DTI were able to directly characterize the cardiomyocytes architecture of diseased myocardium, as opposed to indirectly focusing on scar regions. Further, both imaging modalities were in agreement in demonstrating the loss of transmural helical cardiomyocytes architecture in the presence of disease.

Consistent with previous studies^22,23^, regional analysis comparing peri-infarcted areas to remote areas within ischemic hearts revealed that the impact of ischemia also modifies myocardial orientation in areas not directly affected by the myocardial infarction. Notably, HA in remote areas, where the coronary supply is different from ischemic regions and therefore myocardium is not considered to be influenced by reduced blood supply, were also significantly reduced in comparison to controls. This implies that alteration in cardiomyocytes architecture occurs globally throughout the whole myocardium despite obvious regional differences. This may play an important factor in explaining the reduction of systolic and diastolic myocardial function after myocardial infarction.

The temperature during the DTI acquisition was not actively monitored in the bore. However, the scanner room is temperature controlled to within (20–22 °C) since it is used for *in vivo* animal imaging. Furthermore, we acquired our diffusion measurements in an interleaved fashion such that each diffusion direction is acquired before the next average is acquired as opposed to collecting each diffusion volume sequentially. MD for the whole LV did not significantly change over time.

To date, cardiac biopsy remains the gold standard method with which to identify structural remodeling, including diffuse fibrosis^20,24^, which occurs only at the final stage of cardiac remodeling^25^. Therefore, non-invasive imaging techniques that enable serial monitoring of myocardial remodeling from earlier stage are highly desirable as an alternative to conventional biopsy. Although there have been efforts to non-invasively describe tissue characteristics over disease progression^26,27^, the clinical use of previously reported modalities has been limited due to the requirement for contrast, low spatial resolution, long acquisition time, and risk of radiation exposure. In this regard, cardiac DTI is already an established method for the characterization of myocardial microstructure in animal models^28^. To validate DTI acquired at clinically translatable protocol, and to expand the clinical applicability of DTI in real clinical field, we used 12-diffusion directions to mirror the *in vivo* cardiac DTI protocols^7,8^. DTI may enable earlier detection of the progression of various cardiac diseases and help understanding the structure-function relationship.

### Limitation of the study

The current study is not without limitations. First, the agreement between conventional 2D histology with 3D histology using mirror section was good, but not high. For mirror sectioning, omitting a few slices from each section is inevitable to make surfaces flat. As a result, there was a gap of a few-slices-thick between the two mirror images compared, which can cause disagreement. In addition, a certain degree of tissue swelling is unavoidable during the process of polymerization^11,29^, which can explain both the less than excellent agreement between 2D and 3D histology and the poorer agreement at the smaller transmural depth between DTI and optical images, as cells at the external surface of the heart are more free to expand. However, segmental analysis revealed overall good correlation between DTI and optical images across the entire myocardium. More importantly, established image analysis tools, such as non-rigid co-registration, can be reliably and efficiently used to compare the 3D volumes acquired by 3D histology and DTI across millions of pixels. Second, sheetlet analysis used in several recent studies could not be verified due to the second and third eigenvector of the structure tensor of the cleared tissue was difficult to distinguish. We believe optimized light sheet acquisition via isotopic imaging or improved clearing will hopefully realize better distinction between secondary and tertiary eigenvectors leading to more accurate structure tensor-based sheetlet angle. Like sheetlet angle, HA also reflects the angular orientation of cardiomyocytes architecture, and have been incorporated in both animal and clinical studies^8,28,30^. Lastly, because this study was conducted *ex vivo*, serial imaging to detect temporal changes was not performed, and further study is definitely warranted in this area. In this regard, the current study could serve as a basis for designing and conducting studies using DTI as an indicator of cardiomyocytes microstructure.

## Conclusions

In conclusion, the current study demonstrated that multi-layered helical cardiomyocytes architecture can be ascertained with DTI, by exhibiting concordance with 3D-histology obtained by tissue-clearing technique. By establishing concordance between a novel 3D histology method and DTI in revealing cardiomyocytes architecture, DTI-based characterization of cardiomyocytes orientation represents a promising tool for *in vivo* examination of the microstructural response of the myocardium in various cardiac diseases.

## Materials and Methods

### Mouse model

For the control group, male C57/B6 mice (n = 7) were sacrificed at 12 weeks old to match the ischemic model (n = 8). For the ischemic heart failure model, 8-week-old male C57/B6 mice were anesthetized with 2% isoflurane inhalation using an isoflurane delivery system. After making a small skin incision (1.2 cm) over the chest, the major and minor pectoral muscles were dissected and retracted. The fourth intercostal space was exposed. With a mosquito clamp, a small hole was made at the fourth intercostal space to open the pericardium. The left coronary artery was located and permanently ligated approximately 1 mm from its origin using a 6–0 silk suture. If the anterior wall of the left ventricle became pale, the ligation was considered successful.

The heart was then returned to the intrathoracic space, and the muscle and skin were closed. Mice in ischemic group were recovered in a warm environment. No analgesic was administered during recovery. Mice were checked daily, and body weights were recorded twice a week to monitor the general health of mice until the sacrifice. Ischemic model mice were sacrificed 30 days after surgery to allow for myocardial remodeling. Ischemic heart failure and cardiac remodeling were confirmed by M-mode and 2-dimensional echocardiography immediately before sacrifice.

All procedures were performed in accordance with institutional guidelines and the study was approved by the ethical review board of the Yonsei University for the animals involved in the study.

### Harvesting of the heart and tissue-clearing process using CLARITY

Male C57BL/6 mice were anesthetized with an intraperitoneal injection of zolazepam-tiletamine (30 mg/kg, Zoletil^®^, Virbac) and xylazine (10 mg/kg, Rompun^®^, Bayer Healthcare). Retrograde heart perfusion was then performed on mice with 20 mL of phosphate-buffered saline (PBS) and 20 mL of hydrogel monomer solution (a mixture of 4% [wt/vol] paraformaldehyde [Millipore, 1.04005.1000], 4% [wt/vol] acrylamide [Sigma-Aldrich, A8887], 0.25% [wt/vol] VA-044 [Wako, 017–19362] in PBS). Hearts were extracted and incubated in hydrogel monomer solution at 4 °C for 3 days. Polymerization reaction was carried out by increasing the temperature to 37 °C for 3 hours using an Easy-Gel system (Live Cell Instrument, EG-1001). After polymerization, the heart samples were scanned with DTI.

To clear tissue using the CLARITY technique^11^, heart samples were immersed in pH 8.5 sodium borate buffer containing 4% (wt/vol) SDS (Sigma-Aldrich, L3771), and 100 V was applied across the samples at 37 °C for 3 days, using the Life Canvas incubation system (Live Cell Instrument, EC-1001). After clearing, heart samples were washed in PBS at 37 °C for 2 days. Finally, heart samples were incubated in EZ-index buffer (Live Cell Instrument, EI-Z1001), a custom-made refractive index matching solution with a refractive index of 1.45 in order to make the refractive index of the sample uniform overall, to be optically cleared.

### Optical imaging of the cleared mouse heart

The cleared hearts were scanned using light-sheet fluorescence microscopy, achieving an approximately 5-mm penetration depth. Optical images of clarified intact mouse hearts were mounted with the apex facing the objective lens. The hearts were imaged using a light-sheet fluorescence microscope (Lightsheet Z.1, Carl Zeiss Microscopy Co, Ltd. Germany; stack size, 4.823 mm; 2.283 µm/pixel × 2.283 µm/pixel in-plane resolution; step size 7.67 µm) equipped with a 5× objective (EC Plan-Neofluar 5×) at 638-nm excitation^31^.

### Optical image analysis

Raw optical images were filtered to remove residual stripe-like shadow artifacts^32^. Conventional structure tensor analysis was performed on the filtered images to calculate cardiomyocytes orientation at each voxel^33–35^. Briefly, image gradients (*f*_*x*_*, f*_*y*_*, f*_*z*_) of the optical images were calculated in all dimensions using a Sobel filter to accentuate edges, and image intensity gradients increased the contrast of the cardiomyocytes structure. The following matrices shows the two-dimensional (2D) and 3D structure tensors populated by the image gradients in each dimension defined by (*f*_*x*_*, f*_*y*_*, f*_*z*_) (equations 1 and 2):

1 $$\,2{\rm{D}}:[\begin{array}{c}{f}_{x}{f}_{x}\,{f}_{x}{f}_{x}\\ {f}_{y}{f}_{y}\,{f}_{y}{f}_{y}\end{array}]$$

2 $$\text{3D}:[\begin{array}{c}{f}_{x}{f}_{x}\,{f}_{x}{f}_{y}\,{f}_{x}{f}_{z}\\ {f}_{y}{f}_{x}\,{f}_{y}{f}_{y}\,{f}_{y}{f}_{z}\\ {f}_{z}{f}_{x}\,{f}_{z}{f}_{y}\,{f}_{z}{f}_{z}\end{array}]$$

Eigenvalue decomposition was performed on the structure tensor to yield the secondary eigenvector for 2D imaging or tertiary eigenvector for 3D imaging, which reflects the cardiomyocytes orientation.

### DTI acquisition

After initial perfusion and polymerization, each heart was placed in a 15-ml tube filled with hydrogel-monomer solution. Twelve diffusion-weighted (b = 1000 s/mm^2^) and one non-diffusion-weighted (b = 0 s/mm^2^) single spin echo MRI images^36^ were acquired using a 9.4-T small animal scanner (BioSpec 94/20 USR, Bruker BioSpin) with the same imaging parameters (repetition time = 8750 ms, echo time = 36 ms, number of excitations = 5, spatial resolution = 125 µm × 125 µm × 300 µm, scan time = 14 hours). DTI images were acquired in short-axis orientation, defined as the planes perpendicular to long-axis in both 4- and 2-chamber view, with the most basal image containing at least 50% of circumferential myocardium^37^.

Four-channel surface array RF coil TX/RX was used. Maximum gradient amplitude and slew rate was 440 mT/m and 3440 T/m/s, respectively (diffusion duration = 15 ms, diffusion time = 23 ms, receiver bandwidth = 6000 Hz, number of averages = 10, acquisition matrix = 46 × 92). 2-dimentional acquisition was used with number of slices varied depending on the long axis length of the heart with a few hearts requiring up to 60 slicies at 300 µm slice thickness.

### DTI image analysis

DTI tensor analysis was performed on the acquired diffusion dataset at each voxel using software developed on Matlab (Mathworks, Nattuck, MA)^36,38^. Briefly, a log-linear least squares fit was used to yield the apparent diffusion coefficients (*D*_*xx*_*, D*_*yy*_*, D*_*zz*_*, D*_*xy*_*, D*_*xz*_*, D*_*yz*_) of the self-diffusion tensor defined below (equation 3):

3 $$[\begin{array}{c}{D}_{xx}\,{D}_{xy}\,{D}_{xz}\\ \begin{array}{c}{D}_{xy}\,{D}_{yy}\,{D}_{yz}\\ {D}_{xz}\,{D}_{yz}\,{D}_{zz}\end{array}\end{array}]$$

Eigenvalue decomposition was performed to yield the eigenvectors and eigenvalues. The eigenvector associated with the largest eigenvalue (primary eigenvector) of the estimated self-diffusion tensor at each voxel was assumed to be parallel to the cardiomyocytes orientation^7,39,40^. Helix angle (HA) was calculated using the same geometric definition applied by Streeter *et al*.^1^, with the local tangent vector defined from the center of mass of the left ventricular blood pool to the voxel of interest for each short-axis plane. For 3D visualization, DTI tractography was performed using a FACT algorithm^41^.

### Validation of the integrity of the tissue-clearing technique

To confirm whether the tissue-clearing technique preserves the original cardiomyocytes orientation, we compared the in-plane cardiomyocytes angle, ⊖, calculated from the cleared heart with conventional 2D histological analysis using hematoxylin and eosin (H&E) staining as the gold standard method. As a single tissue cannot undergo two different histologic processes, the comparison was done using the mirror section method^42^. Normal mouse cadaveric hearts were paraffin-embedded and cut into halves at mid-level. The lowest section of the upper part was processed with H&E staining, and the highest section of the lower part was processed with the tissue-clearing technique. The in-plane cardiomyocytes angle was calculated from each section and compared.

In-plane cardiomyocytes angles, ⊖, were calculated using a 2D structure tensor analysis for both conventional histology and tissue-clearing imaging partitions^43^. The cardiomyocytes angles were calculated relative to the axes defined by the following two vectors: vector *v*, defined by the center of masses of the left and right ventricle cavities, and vector *u*, orthogonal to *v*. Regional mean cardiomyocytes angles were compared between conventional histology and tissue-clearing imaging partitions by anatomically segmenting the left ventricular myocardium into 20 radial segments.

### Comparison of myocardial cardiomyocytes architecture between optical images and DTI

HA maps were used to compare cleared 3D optical imaging and DTI. For each heart sample, light-sheet images were down-sampled to match the resolution of DTI. Binary masks were created for both optical and MRI-based HA maps using simple thresholding to identify the myocardium from background.

To compensate for the coarse (1/2600) resolution of DTI (125 × 125 × 300 µm^3^), compared with 3D optical imaging (2.28 × 2.28 × 7.67 µm^3^), 3D tractography of the diffusion tensor data, which subdivided each voxel into 1000 sub-voxels, was used to further qualitatively visualize the helical twisting of the cardiomyocytes from apex to base. Co-registration was performed on the binary masks using a conventional non-rigid, intensity-based mutual information algorithm to obtain a transform matrix that maps the optical binary mask to the MRI binary mask^44^. The transform matrix was applied to the HA maps, and voxel-wise comparison was performed only including voxels for the myocardium (i.e., excluding collagenous scar tissue).

Global HA transmurality (HAT) was calculated for each heart, defined as the mean of the fitted slopes of HA plotted against transmural depth (TD) along 20 equidistant radial projections in the short-axis plane for all slices. The global HAT reflects the degree of helical microstructure present, with lower absolute HAT indicating less helical winding.

### Regional analysis

For control hearts, the equidistant radial projections for all short axis slices used to calculate HAT were binned into 16 AHA segments and averaged within each segment for regional comparison between DTI and light-sheet optical images^45^.

For ischemic hearts, the equidistant radial projections in the remote regions were binned according to the peri-infarct and non-infarcted regions identified by thresholding the mean diffusivity (MD) maps (peri-infarct: 1.12 um^2^/ms > MD > 0.9 um^2^/ms; non-infarct regions: MD <9 um^2^/ms)^22,23^. Infarct regions (MD > 1.12 um^2^/ms) were ignored, as the tensor model for both DTI and light-sheet optical imaging does not adequately characterize the complex tissue microstructure therein^46^. Regional HATs of ischemic hearts were also binned into AHA segments with the infarcted segments omitted.

Further, radial segment analysis, dividing the heart by 3.6° along the axis and hence generating a hundred segments per heart, was used for both controls and ischemic groups.

### Statistical analysis

Continuous variables are presented as mean ± standard error. Means were compared using the non-parametric Wilcoxon test. For per-sample analysis, Bland-Altman analysis was performed to quantify agreement between (1) optical imaging imaging and conventional histology and (2) DTI and optical imaging. Correlations were assessed using Spearman’s correlation coefficient, and ICCanalysis was conducted to examine the relationship between HA calculated from (1) optical imaging and conventional histologic analysis, as well as (2) DTI and optical images. ICC for inter-variability of DTI-determined HA was also calculated.

For regional analysis, correlation between HA values derived from DTI and optical images was calculated using Spearman’s correlation coefficient. For the ischemic model, paired t-test was applied to evaluate differences between peri-infarct areas and remote areas.

All comparisons were two sided, and *p* < 0.05 was considered statistically significant, with 95% confidence intervals also calculated. Statistical analyses were performed using SPSS software, version 23.0 (IBM, Chicago, IL, USA) and MedCalc software, version 16.4.3 (MedCalc Software, Ostend, Belgium).

## Electronic supplementary material

Video 1
